# Associations of lipid profiles with the risk of ischemic and hemorrhagic stroke: A systematic review and meta-analysis of prospective cohort studies

**DOI:** 10.3389/fcvm.2022.893248

**Published:** 2022-11-03

**Authors:** Xiaoxian Gong, Luowei Chen, Bo Song, Xiang Han, Weihai Xu, Bo Wu, Feng Sheng, Min Lou

**Affiliations:** ^1^Department of Neurology, School of Medicine, The Second Affiliated Hospital of Zhejiang University, Hangzhou, China; ^2^Department of Neurology, Zhengzhou University First Affiliated Hospital, Zhengzhou, China; ^3^Department of Neurology, Huashan Hospital, Fudan University, Shanghai, China; ^4^Department of Neurology, State Key Laboratory of Complex Severe and Rare Diseases, Peking Union Medical College Hospital, Chinese Academy of Medical Sciences and Peking Union Medical College, Beijing, China; ^5^Department of Neurology, West China Hospital, Sichuan University, Chengdu, China; ^6^Medical Development, Amgen Biology Technology Consulting (Shanghai) Co., Ltd., Shanghai, China

**Keywords:** ischemic stroke, hemorrhagic stroke, total cholesterol, triglyceride, high-density lipoprotein cholesterol, low-density lipoprotein cholesterol

## Abstract

**Background and purpose:**

The associations of lipid profiles with the risk of ischemic stroke (IS) or hemorrhagic stroke (HS) are controversial. In this study, we aimed to illustrate the optimal level for lipid levels in the risk of IS and HS.

**Materials and methods:**

We searched the electronic database of PubMed, Embase, and the Cochrane library from inception until November 2020. Prospective cohort studies published in English for the associations of lipid profiles (TC, TG, LDL-C, HDL-C, and non–HDL-C) with the risk of IS and HS were eligible for this study, and the publication status was not restricted. We calculated the pooled effect estimates using the random-effects model. We tested the associations of lipid profiles with IS and HS and compared their differences.

**Results:**

We retrieved 50 prospective cohort studies containing 3,301,613 individuals. An increase in total cholesterol (TC) is associated with an increased IS risk (*P* < 0.001) and a reduced HS risk (*P* < 0.001). Similarly, an increase in triglyceride links with a greater IS risk (*P* < 0.001) but with a lower HS risk (*P* = 0.014). On the opposite, high-density lipoprotein cholesterol (HDL-C) correlates with a reduced IS risk (*P* = 0.004) but has no significant association with the HS risk (*P* = 0.571). Moreover, an increase in low-density lipoprotein cholesterol (LDL-C) or non–high-density lipoprotein cholesterol has no statistically significant effect on both IS and HS. The pooled effect estimates on the risk of IS and HS revealed that TC and LDL-C levels should be controlled under 6.0 and 3.5 mmol/L, respectively, to reduce worsening effects on the IS risk while maintaining potential beneficial effects on reducing the HS risk.

**Conclusion:**

We revealed comprehensive relationships between lipid profiles and the risk of stroke, suggesting controlling the TC and LDL-C levels under 6.0 and 3.5 mmol/L, respectively, to balance both the IS and HS risk.

## Introduction

The effects of lipid profiles on the risk of ischemic stroke (IS) or hemorrhagic stroke (HS) have been evaluated in several systematic reviews and meta-analyses; however, they did not clearly clarify the distinctive role of lipid profiles in IS and HS (1, 2). A nest case-control study of China Kadoorie Biobank found there were positive associations of LDL-C with IS and inverse associations with HS, which were confirmed by genetic analyses and LDL-C-lowering trials (3). Therefore, it becomes an important issue to determine an optimal range of lipid profiles to balance their benefits and harmful effects on IS and HS. Thus, we conducted a large-scale examination of prospective cohort studies to evaluate how lipid profiles are associated with IS and HS risks in the general population. Especially, we aimed to compare the optimal ranges of lipid profiles on the risk of IS and HS by an indirect approach.

## Methods

### Data sources, search strategy, and selection criteria

We conducted this study following the Meta-analysis of Observational Studies in Epidemiology protocol (4). We searched the electronic databases of PubMed, Embase, and Cochrane Library from inception until November 2020, by using the text word or Medical Subject Heading (MeSH) of the following search terms: (“cerebral hemorrhage” OR “subarachnoid Hemorrhage” OR “hemorrhagic stroke” OR “cerebral infarction” OR “ischemic stroke”) AND (“cholesterol” OR “hypercholesterolemia” OR “dyslipidemia” OR “lipid profile” OR “triglycerides” OR “low-density lipoprotein” OR “high-density lipoprotein” OR “non-high-density lipoprotein”). We also hand-searched the references listed in the potentially relevant literature to select additional eligible studies.

Two authors (XG and LC) independently searched for literature and assessed the obtained trials following the standardized approach.

We discussed and settled conflicts until reaching a consensus (XG, LC, and ML). The following are the criteria for the inclusion of the publications: (1) all subjects were initially free of IS and HS; (2) appropriate exposure measures for lipid profiles included TC, TG, LDL-C, HDL-C, and non–HDL-C; (3) outcomes included the incidence of IS and HS; and (4) the effect estimates and 95% confidence intervals (CIs) were used to compare lipid profiles versus minimal lipid profiles categories. Furthermore, if the study reported several multivariable-adjusted effects estimates, we selected the effect estimates with maximally adjusted covariates and excluded retrospective cohort and case-control studies to avoid uncontrolled confounders biasing the results.

### Data collection and quality assessment

Two researchers (BS and XH) evaluated the included studies and extracted the data, and an additional author (ML) checked the extracted data independently. We considered the covariates adjusted only for age and sex as low adjusted levels. Two authors assessed the study quality by using the Newcastle–Ottawa Scale (NOS) (5). We considered the staring system of NOS ranging from 0 to 9 and studies with 7 to 9 stars as high quality. A third author reviewed the original articles independently and adjudicated disagreement if any.

### Statistical analysis

We analyzed the roles of lipid profiles with the risk of IS and HS based on the effect estimates [odds ratio (OR), relative risk (RR), and hazard ratio (HR)] and their 95% CIs in each individual study. We used the generalized least-squares method for trend estimation to calculate the category-specific risk estimates, in which the relative risk (RR) is associated with an increase of 1 mmol/L in lipid profiles (6), and these estimates were calculated based on the assumption that a linear and non-linear relationship is present between the natural logarithm of the RR and the increasing lipid profiles levels. We assumed that the distribution of lipid profiles met normality and assigned the median concentration of lipid profiles (the mid-point for closed categories or the median for open categories) to the corresponding RR. The random-effects model was used to calculate the high (TC: > 6.0; TG: > 3.0; LDL-C: > 4.0; HDL-C: > 1.8; non–HDL-C: > 5.0 mmol/L), moderate (TC: 5.0–6.0; TG: 2.0–3.0; LDL-C: 3.5–4.0; HDL-C: 1.5–1.8; non–HDL-C: 4.0–5.0 mmol/L), and light (TC: < 5.0; TG: < 2.0; LDL-C: < 3.5; HDL-C: < 1.5; non–HDL-C: < 4.0 mmol/L) levels versus the lowest categories of lipid profiles in individual studies, and the risk of IS and HS associated with an increase of lipid profiles by 1 mmol/L (7). The cut-off point of lipid parameters for high, moderate, and light referred to the quartiles distribution of lipid profiles. The indirect comparisons were used to compare the potential beneficial and harmful effects of lipid profiles on IS and HS (8). We derived the dose–response curve from the modeled lipid profiles using the restricted cubic splines with knee knots at fixed percentiles of 10, 50, and 90% of the distribution (6), and also extracted the distribution of case numbers, persons or person-years, and effect estimates with 95% CIs for ≥ 3 quantitative exposure categories.

We assessed heterogeneity across the studies using the *I*^2^ and Q statistic and defined significant heterogeneity as *I^2^* > 50.0% or *P* < 0.10 (9). We performed a one-by-one sensitivity analysis to estimate the effects of individual studies on the overall meta-analysis results (10) and then analyzed the subgroups based on the country, sex, follow-up duration, and adjusted levels. Several methods, including funnel plots for qualitative and Egger and Begg test, were used for quantitative measurements (11, 12) to compute potential publication bias. The inspection levels for pooled results were two-sided, and *P* < 0.05 was regarded as statistically significant. All analyses in this study were carried out using STATA software (version 10.0; Stata Corporation, College Station, TX, USA).

## Results

### Literature search

We initially identified 2,365 articles and retained 924 articles after removing duplicate titles, and further excluded 815 articles owing to irrelevant topics. The remaining 109 studies were retrieved for further full-text evaluations, and 59 articles were excluded due to their retrospective design (*n* = 34), or not separately reported for IS or HS (*n* = 15), or they were review or meta-analysis articles (*n* = 10). As a result, we retrieved 50 studies for meta-analysis. The manual search yielded no additional eligible study (Supplementary File 2). The baseline characteristics of the studies and participants included were summarized (Supplementary Table 1).

### Study characteristics

The final meta-analysis involved a total of 3,301,613 individuals in the 50 studies, in which 28 studies were conducted in Europe or the USA, while the remaining 22 studies were conducted in Asia. The follow-up duration ranged from 2.7 to 38.0 years. Lipid profiles were associated with the IS risk in 40 cohorts and with the HS risk in 35 cohorts. We determined study quality by the NOS, by which the following are the results: 16 cohorts had nine stars, 20 cohorts had eight stars, 13 cohorts had seven stars, and the remaining one study contained cohorts ranging from 7 to 9 stars (Supplementary File 1).

### Dose–response meta-analysis

**FIGURE 1:**
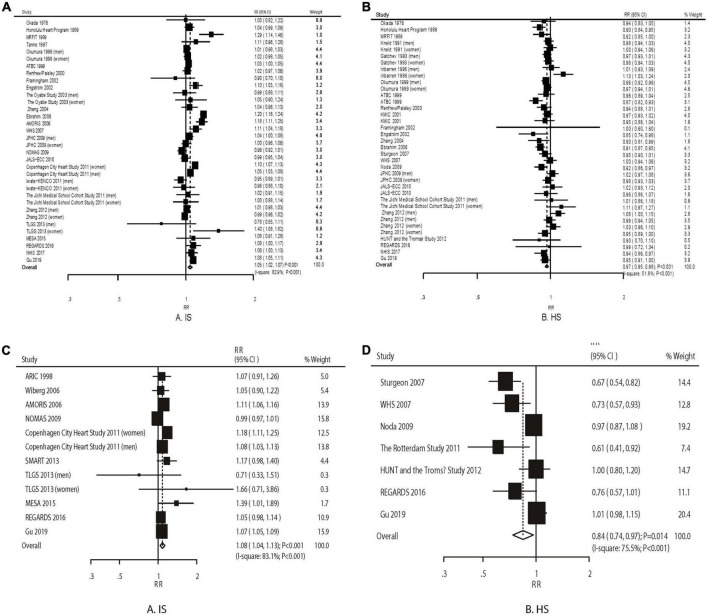
The dose–response meta-analysis per 1 mmol/L increment in TC for ischemic **(A)** and hemorrhagic stroke **(B)**. The dose–response meta-analysis per 1 mmol/L increment in TG for ischemic **(C)** and hemorrhagic stroke **(D)**.

The pooled results suggested that a 1 mmol/L increment in TC level was associated with an increased risk of IS (RR: 1.05; 95% CI: 1.02–1.07; *P* < 0.001) and a reduced risk of HS (RR: 0.97; 95% CI: 0.95–0.98; *P* < 0.001). We observed a significant heterogeneity across the included cohorts for IS (*I*^2^ = 82.9%; *P* < 0.001) and HS (*I*^2^ = 51.8%; *P* < 0.001) (Figures 1A,B). These conclusions were robust as evaluated by the sequential exclusion of individual studies (Supplementary File 3).

An increment in TG by 1 mmol/L was associated with an increased risk of IS (RR: 1.08; 95% CI: 1.04–1.13; *P* < 0.001) and a reduced risk of HS (RR: 0.84; 95% CI: 0.74–0.96; *P* = 0.010) (Figures 1C,D). The heterogeneity was significant for IS (*I*^2^ = 83.1%; *P* < 0.001) and HS (*I*^2^ = 75.5%; *P* < 0.001). The conclusions from the pooled analyses for IS were consistent yet varied for HS (Supplementary File 3).

**FIGURE 2:**
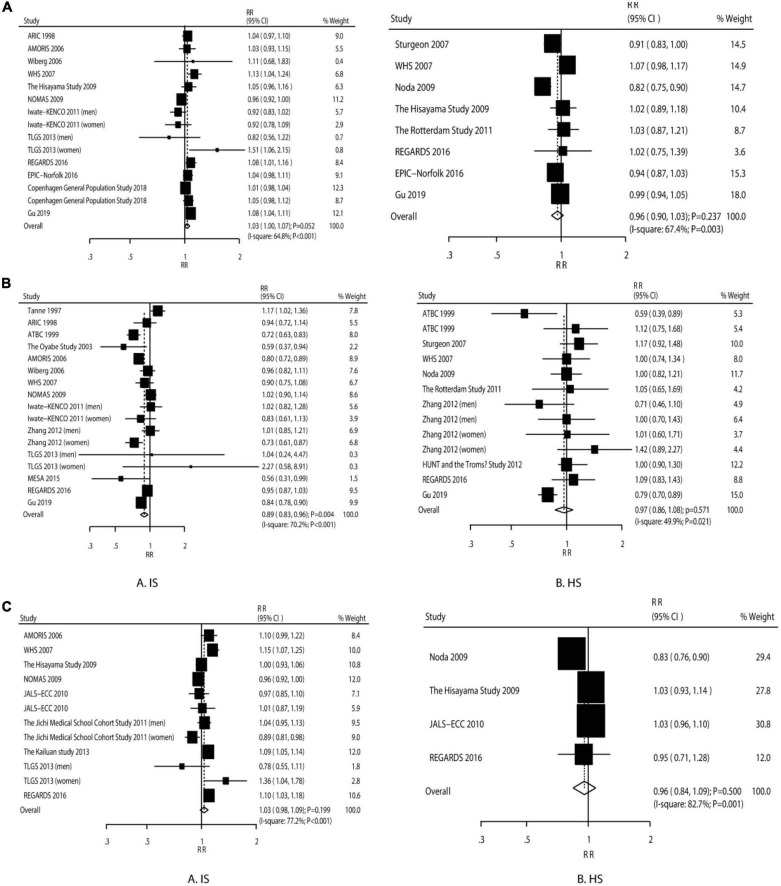
The dose–response meta-analysis per 1 mmol/L increment in LDL for ischemic and hemorrhagic stroke **(A)**. The dose–response meta-analysis per 1 mmol/L increment in HDL for ischemic and hemorrhagic stroke **(B)**. The dose–response meta-analysis per 1 mmol/L increment in non–HDL for ischemic and hemorrhagic stroke **(C)**.

The IS risk (RR: 1.03; 95% CI: 1.00–1.07; *P* = 0.052) and HS risk (RR: 0.96; 95% CI: 0.90–1.03; *P* = 0.237) were not significantly increased by 1 mmol/L of LDL (Figure 2A). We observed a significant heterogeneity among the included studies (IS: *I*^2^ = 64.8%, *P* < 0.058; HS: *I*^2^ = 67.4%; *P* = 0.003). The sensitivity analysis indicated that the pooled analysis conclusions for IS and HS were weak (Supplementary File 3).

The IS risk was significantly reduced (RR: 0.89; 95% CI: 0.83–0.96; *P* = 0.004), while the HS risk was not affected by an increment in HDL-C by 1 mmol/L (RR: 0.97; 95% CI: 0.86–1.08; *P* = 0.571) (Figure 2B). There was a significant heterogeneity for IS (*I*^2^ = 70.2%; *P* < 0.001) and HS (*I*^2^ = 49.9%; *P* = 0.021). The sensitivity analyses suggested that both the pooled analysis conclusions for IS and HS were robust (Supplementary File 3).

The increment in non–HDL-C by 1 mmol/L was not associated with the IS risk (RR: 1.03; 95% CI: 0.98–1.09; *P* = 0.199) and HS risk (RR: 0.96; 95% CI: 0.84–1.09; *P* = 0.500) (Figure 2C). There was significant heterogeneity across the studies for IS (*I*^2^ = 77.2%; *P* < 0.001) and HS (*I*^2^ = 82.7%; *P* = 0.001). The sensitivity analyses suggested firm conclusions (Supplementary File 3).

### Different categories versus the lowest lipid profiles

First, the high TC levels yield excessive IS risk, but no significant association was found between the IS risk and the moderate as well as light TC levels (Table 1). High, moderate, and light TC levels were associated with a reduced risk of HD. Nevertheless, high TC induced excessive IS risk than moderate or light TC, while no significant difference exists for HS. These results suggest that the TC levels could be better controlled under 6.0 mmol/L (the lower bound of the high TC level) in order to balance the risks of IS and HS. Second, high, moderate, and light TG levels are associated with an increased risk of IS, while the HS risk is significantly reduced when the TG levels are in the moderate and light TG levels. High TG was associated with an increased risk of IS. Therefore, the level of TG should be controlled under 3.0 mmol/L to balance the risks of IS and HS. Third, high LDL-C was associated with an increased risk of IS, while light LDL-C levels could protect against HS risk. These results suggested that the LDL-C level could be better controlled under 3.5 mmol/L. Fourth, high and light levels of HDL-C could protect against the risk of IS, while HDL-C levels have no significant association with the HS risk. The moderate non–HDL-C level was associated with an increased risk of IS, while the non–HDL-C level is not associated with the risk of HS.

**TABLE 1:** The pooled effect estimates for various categories of lipid profiles on the risk of ischemic and hemorrhagic stroke.

Lipid profiles	Outcomes	Subgroup	RR and 95% CI	*P*-value	Heterogeneity (%)	*P*-value for heterogeneity	High versus moderate for IS/HS	High versus light for IS/HS	Moderate versus light for IS/HS
TC	IS	> 6.0 mmol/L (high)	1.29 (1.16–1.43)	< 0.001	70.3	< 0.001	1.21 (1.05–1.38)/	1.21 (1.02–1.43)/	1.00 (0.85–1.17)/
	HS	> 6.0 mmol/L (high)	0.81 (0.74–0.90)	< 0.001	46.2	< 0.001	0.95 (0.82–1.10)	0.98 (0.81–1.17)	1.02 (0.85–1.24)
	IS	5.0–6.0 mmol/L (moderate)	1.07 (0.98–1.16)	0.153	39.6	0.016			
	HS	5.0–6.0 mmol/L (moderate)	0.85 (0.76–0.94)	0.002	30.5	0.047			
	IS	< 5.0 mmol/L (light)	1.07 (0.94–1.23)	0.312	0.0	0.859			
	HS	< 5.0 mmol/L (light)	0.83 (0.71–0.97)	0.019	24.4	0.156			
TG	IS	> 3.0 mmol/L (high)	1.55 (1.34–1.79)	< 0.001	0.0	0.494	1.15 (0.90–1.47)/	1.36 (1.14–1.62)/	1.18 (0.95–1.48)/
	HS	> 3.0 mmol/L (high)	0.98 (0.63–1.54)	0.945	0.0	0.471	1.72 (0.89–3.33)	1.21 (0.74–1.97)	0.70 (0.42–1.19)
	IS	2.0–3.0 mmol/L (moderate)	1.35 (1.11–1.66)	0.003	75.2	0.003			
	HS	2.0–3.0 mmol/L (moderate)	0.57 (0.35–0.93)	0.025	80.7	< 0.001			
	IS	< 2.0 mmol/L (light)	1.14 (1.03–1.26)	0.010	24.6	0.233			
	HS	< 2.0 mmol/L (light)	0.81 (0.67–0.99)	0.036	44.2	0.073			
LDL	IS	> 4.0 mmol/L (high)	1.16 (1.05–1.29)	0.004	26.9	0.180	1.10 (0.94–1.30)/	1.22 (0.99–1.50)/	1.11 (0.89–1.38)/
	HS	> 4.0 mmol/L (high)	0.79 (0.60–1.06)	0.118	64.8	0.006	0.85 (0.62–1.16)	1.25 (0.87–1.81)	1.48 (1.14–1.91)
	IS	3.5–4.0 mmol/L (moderate)	1.05 (0.92–1.19)	0.498	52.5	0.049			
	HS	3.5–4.0 mmol/L (moderate)	0.93 (0.82–1.05)	0.238	0.0	0.483			
	IS	< 3.5 mmol/L (light)	0.95 (0.80–1.14)	0.598	0.0	0.505			
	HS	< 3.5 mmol/L (light)	0.63 (0.50–0.79)	< 0.001	22.9	0.262			
HDL	IS	> 1.8 mmol/L (high)	0.73 (0.55–0.98)	0.034	72.3	0.003	0.86 (0.61–1.20)/	0.89 (0.65–1.22)/	1.04 (0.84–1.28)/
	HS	> 1.8 mmol/L (high)	0.97 (0.68–1.38)	0.877	59.3	0.031	0.91 (0.58–1.41)	1.04 (0.69–1.57)	1.15 (0.82–1.61)
	IS	1.5–1.8 mmol/L (moderate)	0.85 (0.72–1.02)	0.076	50.8	0.058			
	HS	1.5–1.8 mmol/L (moderate)	1.07 (0.82–1.40)	0.634	23.1	0.267			
	IS	< 1.5 mmol/L (light)	0.82 (0.73–0.93)	0.002	38.2	0.066			
	HS	< 1.5 mmol/L (light)	0.93 (0.76–1.14)	0.486	28.5	0.131			
Non–HDL	IS	> 5.0 mmol/L (high)	1.10 (0.75–1.63)	0.618	74.1	0.004	0.78 (0.47–1.30)/	1.05 (0.67–1.64)/	1.34 (0.90–2.01)/
	HS	> 5.0 mmol/L (high)	0.74 (0.44–1.27)	0.277	61.8	0.049	1.25 (0.36–4.41)	1.00 (0.54–1.84)	0.80 (0.24–2.60)
	IS	4.0–5.0 mmol/L (moderate)	1.41 (1.01–1.97)	0.044	68.9	0.022			
	HS	4.0–5.0 mmol/L (moderate)	0.59 (0.19–1.86)	0.366	88.8	0.003			
	IS	< 4.0 mmol/L mmol/L (light)	1.05 (0.84–1.32)	0.664	59.3	0.022			
	HS	< 4.0 mmol/L mmol/L (light)	0.74 (0.55–1.01)	0.060	56.1	0.025			

RRI, relative risk increase; RR, relative risk; CI, confidence interval; IS, ischemic stroke; HS, hemorrhagic stroke.

### Dose–response curve

We found significant non-linear relationships between TC with the IS (*P* < 0.001) and the HS risk (*P* = 0.001). Similar significant non-linear relationships exist for (1) TG with the IS (*P* < 0.001); (2) TG with the HS risk (*P* = 0.014); (3) LDL-C with the IS (*P* = 0.003); and (4) HDL-C and IS (*P* = 0.003). However, such relationships do not exist for (1) LDL-C with HS (*P* = 0.066); (2) HDL-C with HS (*P* = 0.238); (3) non–HDL-C and IS (*P* = 0.375); and (4) non–HDL-C and HS (*P* = 0.574) (Supplementary File 4).

### Subgroup analyses in various populations

Studies performed in Western countries suggest an association between an increment in TC by 1 mmol/L with an increased risk of IS, while those conducted in Eastern countries showed that an increment in TC by 1 mmol/L could protect against HS. An increment in TG by 1 mmol/L was associated with an increased risk of IS and a reduced HS risk in Western countries. Similar to TG, an increment in LDL-C by 1 mmol/L was associated with higher IS and lower HS risks. An increment in HDL-C by 1 mmol/L was linked with reduced IS risks. Apart from these observations, we did not find other significant associations of lipid profiles with the risk of IS and HS (Supplementary Table 2).

### Publication bias

We found no significant publication biases for the IS risk with the increment by 1 mmol/L in TC, TG, LDL-C, HDL-C, and non–HDL-C, and for the HS risk associated with TC, LDL-C, HDL-C, and non–HDL-C (Supplementary File 5). We observed potential publication bias for the association between TG and HS (*P*-value for Egger: 0.018; *P*-value for Begg: 0.072). This conclusion was not altered by the adjusted publication bias using the trim and fill method (13).

## Discussion

We updated the previous meta-analyses’ results. An increase in TC is associated with the IS risk, but it may also play a protective role in HS. Excessive TC impacts on inflammatory atherogenic lipid molecules lead to a high IS risk (14). Nevertheless, low TC levels might promote cell necrosis of the arterial medial layer, which is susceptible to microaneurysms and related to the HS onsets (15). Of note, the pooled effect estimates suggest that the high TC level is associated with an increased IS risk, while high, moderate, and light TC levels could protect against HS risk, deriving a cut-off value under 6.0 mmol/L.

We also found that an increase in TG was significantly associated with an IS risk, but it may be beneficial for HS. In addition to a direct atherogenic effect of triglyceride-rich lipoproteins, high TG appears to be a marker for a series of other potentially atherogenic and prothrombotic changes. Furthermore, elevated triglycerides are associated with several abnormalities of the clotting–fibrinolytic systems (16), which may contribute further to their association with IS. Because the relationship between TG and HS appeared essentially along with the increased TC level, the beneficial impact of the increased TG level on the HS might indicate synergic effects between TC and TG on HS occurrence (17).

The increment in LDL-C by 1 mmol/L does not affect the risk of IS and HS, but the pooled analysis conclusion is marginal, as the high LDL-C still yields a harmful effect on IS, while the light LDL-C constitutes a protective role on the risk of HS. Notably, LDL-C is a major target for lowering IS risk; virtually all national and international guidelines for IS prevention and treatment guidelines recommendation lowering LDL-C. Nevertheless, the results on the relationship between LDL-C and HS are inconsistent. Although some studies found no relationship between LDL-C and HS (18–21), other studies found LDL-C to be negatively associated with HS (22). Indeed, a lower LDL-C might lead to arterionecrosis, resulting in arterial fragility (23). Moreover, LDL-C may promote platelet activation and tissue factor expression, leading to impaired coagulation function (24). Therefore, our results suggest controlling the level of LDL under 3.5 mmol/L to balance the risks of IS and HS, given the hazard ratios for HS in those with moderate LDL-C were significantly higher when compared with participants with light LDL-C.

For participants with an increased HDL-C level, a lower IS risk was found, while no significant association with HS was verified. A potential reason is that HDL-C could reverse the cholesterol transport and lead to a rapid clearance of the cholesteryl esters, which could prevent atherosclerotic progression (25). Moreover, the HS prevalence was lower than expected, and the power might not be enough to detect potential significant relationships.

Our subgroup analyses showed that a 1 mmol/L increase in TC was associated with a high IS risk and a low HS risk in men but not in women. The steep decline in estrogen corresponds well with the sharp increase in TC, which could contribute to the association of TC with the risk of IS and HS. Moreover, the increment of 1 mmol/L in TG was associated with increased IS risk and reduced HS risk in the western countries, but not in the eastern countries. First, non-uniform risk stratification on the risk of IS and HS exists between Eastern and Western countries (26). Second, the distribution of traditional cardiovascular risk factors (e.g., systolic blood pressure, current smoking, and drinking) differs when the population is stratified by sex (27). Third, the high and low adjusted results could affect the results’ reliability, as various traditional cardiovascular risk factors affect stroke occurrence. Fourth, the number of cohorts in each subdivision could affect the stability of pooled studies’ conclusions, which requires to be verified by further large-scale prospective studies.

We stress a few strengths of our meta-analysis. (1) We conducted the analysis based on prospective cohort studies which could minimize recall biases in retrospective studies. (2) The analysis was based on a large sample size. Thus, the results were robust. (3) We compared the IS and HS risk related to the various categories of lipid profiles generating potential optimal lipid levels. (4) We conducted sensitivity and subgroup analysis to explore the sources of heterogeneity.

Nevertheless, we cannot ignore our study’s limitations. First, we were unable to fully explain the substantial heterogeneity across the included studies by sensitivity and subgroup analysis. Second, the number of cohorts investigating the roles of TG, LDL-C, HDL-C, and non–HDL-C was relatively small, and the results of stratified analysis varied. Third, the adjusted covariates changed among the included studies, which might affect the associations between lipid profiles and the risk of IS and HS. Fourth, the differences between subgroups were calculated based on indirect comparisons, and the results’ reliability requires verification.

In summary, we revealed comprehensive relationships between lipid profiles and stroke risks, suggesting controlling the TC and LDL-C levels under 6.0 and 3.5 mmol/L, respectively, to balance IS and HS risks. In addition, we showed that sex and geological regions could affect the associations of different lipid profiles with the risk of IS and HS. We deem that further large-scale prospective studies should be conducted to verify our findings.

## Data availability statement

The raw data supporting the conclusions of this article will be made available by the authors, without undue reservation.

## Author contributions

ML conceived and coordinated the study. XG, LC, and FS carried out the data collection and data analysis. XG and LC wrote the manuscript. ML, BS, XH, WX, and BW revised the manuscript. All authors reviewed the results and approved the final version of the manuscript.
