# Supplementary material for: Associations of lipid profiles with the risk of ischemic and hemorrhagic stroke: A systematic review and meta-analysis of prospective cohort studies
Source: Front Cardiovasc Med. 2022 Nov 3;9:893248. doi: 10.3389/fcvm.2022.893248 (PMC9668898; doi:10.3389/fcvm.2022.893248)
Supplement: Supplementary file 1 [file Data_Sheet_1.ZIP › 893248_SupMaterial/S4 File.DOCX]

**
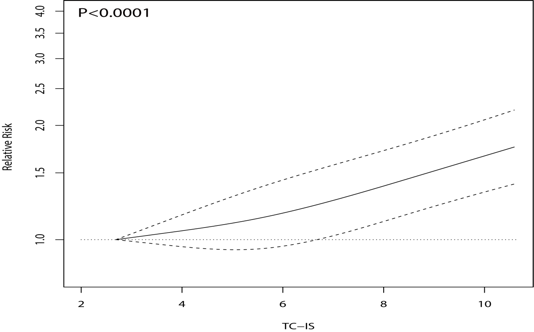
**

**Figure S1. Dose-response relations between TC and relative risks of ischemic stroke**

**
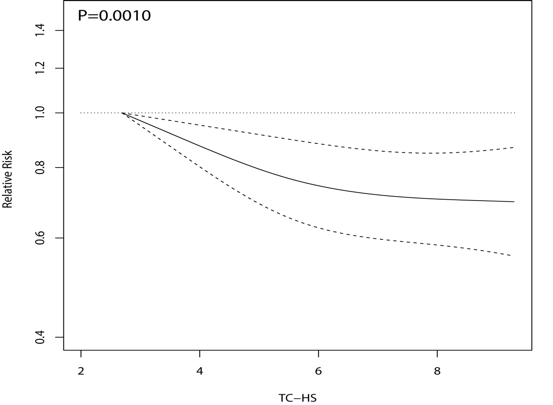
**

**Figure S2. Dose-response relations between TC and relative risks of hemorrhagic stroke**

**
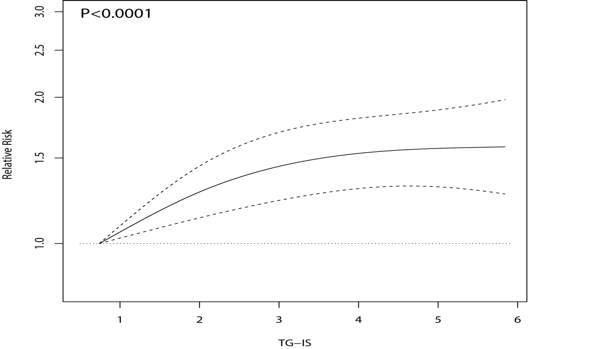
**

**Figure S3. Dose-response relations between TG and relative risks of ischemic stroke**

**
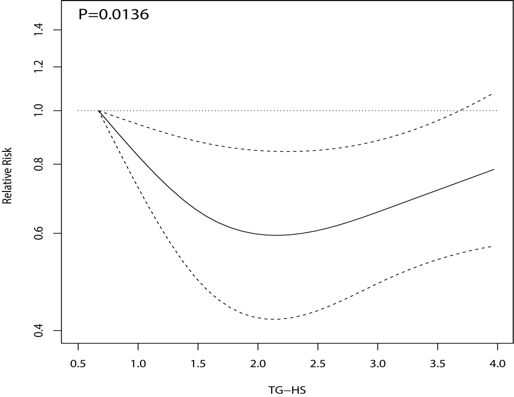
**

**Figure S4. Dose-response relations between TG and relative risks of hemorrhagic stroke**

**
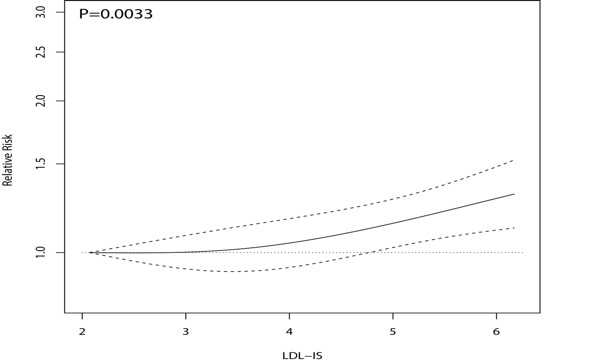
**

**Figure S5. Dose-response relations between LDL and relative risks of ischemic stroke**

**
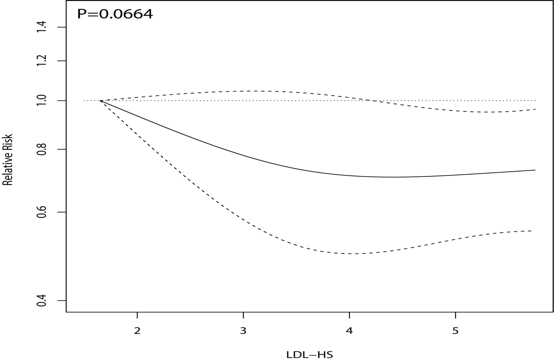
**

**Figure S6. Dose-response relations between LDL and relative risks of hemorrhagic stroke**

**
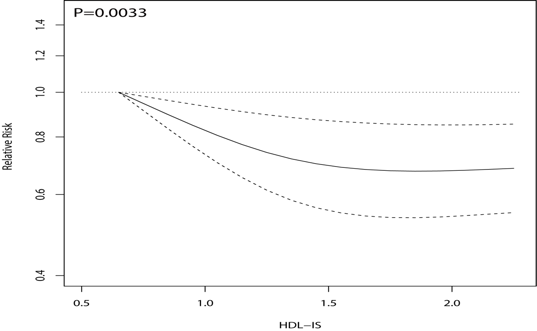
**

**Figure S7. Dose-response relations between HDL and relative risks of ischemic stroke**

**
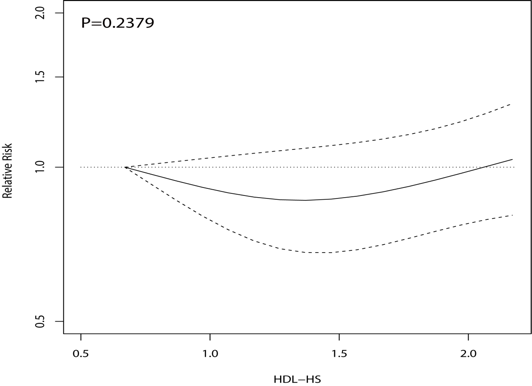
**

**Figure S8. Dose-response relations between HDL and relative risks of hemorrhagic stroke**

**
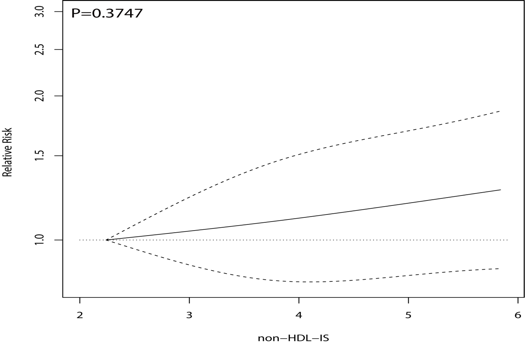
**

**Figure S9. Dose-response relations between non-HDL and relative risks of ischemic stroke**

**
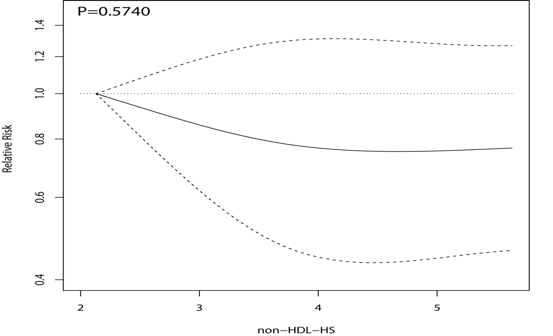
**

**Figure S10. Dose-response relations between non-HDL and relative risks of hemorrhagic stroke**
