# Supplementary material for: Associations of lipid profiles with the risk of ischemic and hemorrhagic stroke: A systematic review and meta-analysis of prospective cohort studies
Source: Front Cardiovasc Med. 2022 Nov 3;9:893248. doi: 10.3389/fcvm.2022.893248 (PMC9668898; doi:10.3389/fcvm.2022.893248)
Supplement: Supplementary file 1 [file Data_Sheet_1.ZIP › 893248_SupMaterial/S5 File.DOCX]

**
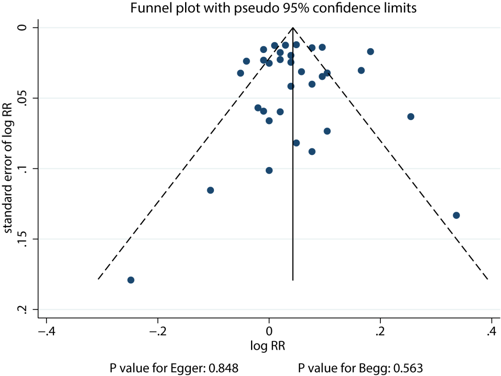
**

**Figure S1. Publication bias for the association between TC and the risk of IS**

**
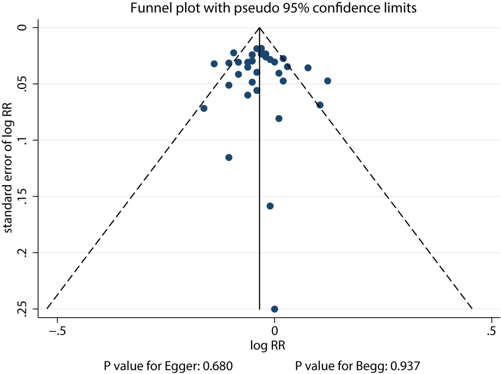
**

**Figure S2. Publication bias for the association between TC and the risk of HS**

**
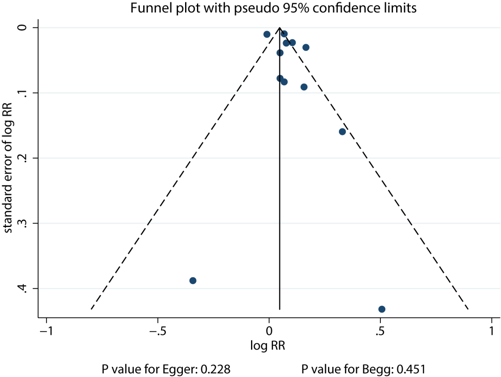
**

**Figure S3. Publication bias for the association between TG and the risk of IS**

**
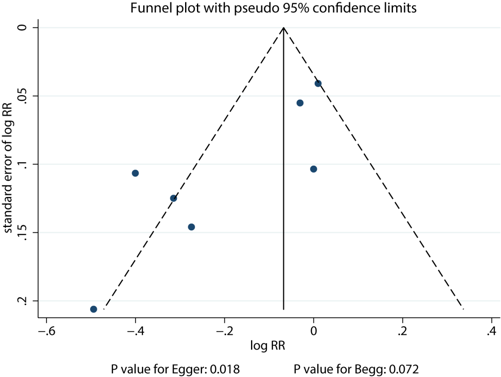
**

**Figure S4. Publication bias for the association between TG and the risk of HS**

**
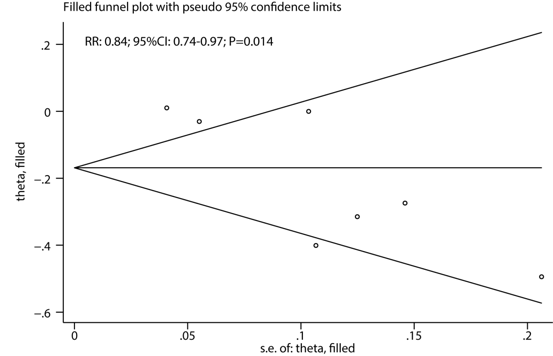
**

**Figure S5. The association between TG and the risk of HS adjusted by using the trim and fill method**

**
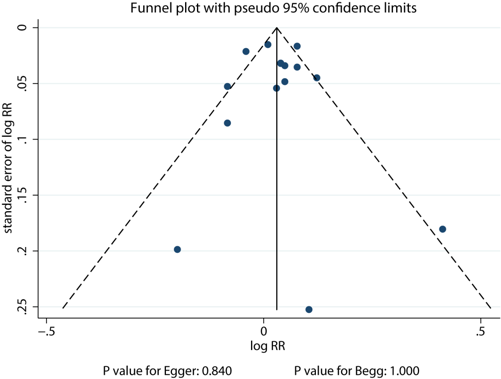
**

**Figure S6. Publication bias for the association between LDL and the risk of IS**

**
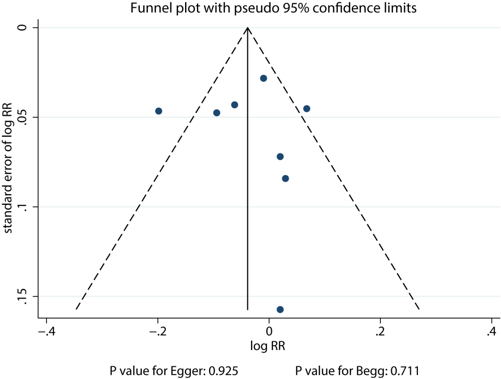
**

**Figure S7. Publication bias for the association between LDL and the risk of HS**

**
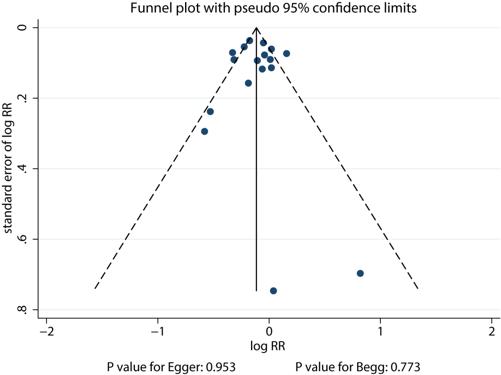
**

**Figure S8. Publication bias for the association between HDL and the risk of IS**

**
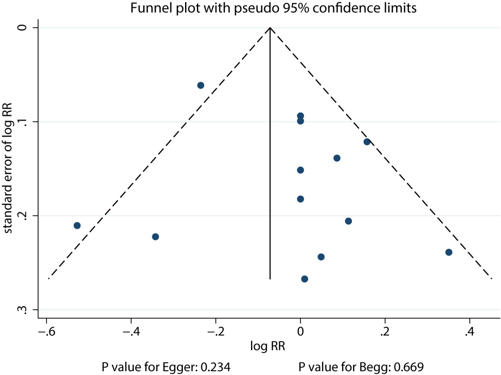
**

**Figure S9. Publication bias for the association between HDL and the risk of HS**

**
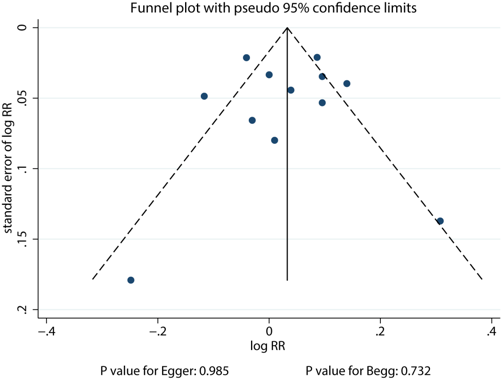
**

**Figure S10. Publication bias for the association between non-HDL and the risk of IS**

**
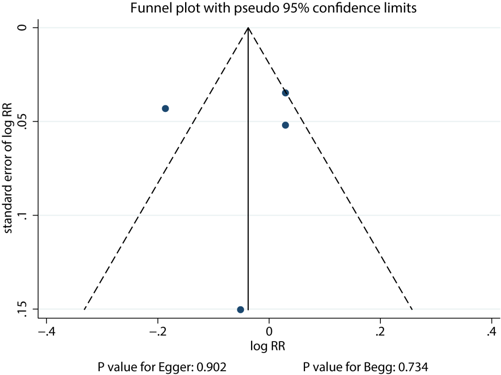
**

**Figure S11. Publication bias for the association between non-HDL and the risk of HS**
