# Supplementary material for: Associations of lipid profiles with the risk of ischemic and hemorrhagic stroke: A systematic review and meta-analysis of prospective cohort studies
Source: Front Cardiovasc Med. 2022 Nov 3;9:893248. doi: 10.3389/fcvm.2022.893248 (PMC9668898; doi:10.3389/fcvm.2022.893248)
Supplement: Supplementary file 1 [file Data_Sheet_1.ZIP › 893248_SupMaterial/supplementary Table 1.DOCX]

**supplementary Table 1. The characteristics of identified studies and participants**

| Study | Country | Sample size | Age (years) | Men (%) | Exposure | Reported outcomes | Effect  estimate | Comparison categories (mmol/L) | Follow-up (years) | Covariates in fully adjusted model | Study quality |
| --- | --- | --- | --- | --- | --- | --- | --- | --- | --- | --- | --- |
| Okada 1976(1) | Japan | 4,737 | 40-79 | 46.4 | TC | IS (51); HS (58) | RR | > 5.69; 4.91-5.69; 4.14-4.91; 3.36-4.14; <3.36 | 6.0 years | Age, and sex | 8 |
| Honolulu Heart Program 1989(2) | Japan | 7,850 | 45-68 | 100.0 | TC | HS (116); IS (330) | RR | Lowest quintile (1.32- 4.87) vs higher quintiles (>4.87) | 18.0 years | Age, DBP, serum UA, cigarettes/d, and alcohol consumption | 9 |
| MRFIT 1989(3, 4) | US | 350,977 | 35-57 | 100.0 | TC | IS (92); HS (138) | RR | >7.23; 6.19-7.22; 5.16-6.18; 4.14-5.15; <4.14 | 6.0 and 12.0 years | Age, DBP, cigarettes/d, and race | 9 |
| Knekt 1991(5) | Finland | 42,862 | 20-69 | 54.0 | TC | HS (187) | RR | ≤5.96; 5.98-6.99; >6.99 | 12.0 years | Age | 8 |
| Gatchev 1993(6) | Sweden | 54,385 | 25-74 | 49.0 | TC | Fatal HS (434) | RR | Lowest quintile vs higher quintiles | 20.5 years | Age, follow-up period, and DBP | 9 |
| Iribarren 1996(7) | US | 61,756 | 20-89 | 46.0 | TC | HS (386) | RR | Levels below 10^th^ percentile vs higher levels | 10.7 years | Age, race, education level, BMI, SBP, smoking, alcohol intake, blood glucose, and prevalent medical conditions | 9 |
| Tanne 1997(8) | Israel | 8,586 | >42 | 100.0 | TC, HDL | Fatal IS (295) | RR | Per 40 mg/dL, and 10 mg/dL increase | 21.0 years | Age, BMI, DM, TC, SBP, history of CHD, and smoking | 8 |
| ARIC 1998(9-11) | US | 14,175 | 45-64 | 44.5 | LDL, HDL, TG | IS (305) | HR | <25^th^;25^th^-25^th^;50^th^-74^th^;>25^th^ | 10.0 years | Age, sex, race-field center, SBP, use of antihypertensive medications, smoking, DM, left ventricular hypertrophy by ECG, plasma levels of fibrinogen and von Willebrand factor, and education level | 8 |
| Okumura 1999(12) | Japan | 38,053 | 33-93 | 47.0 | TC | HS (120), IS (164) | OR | ≤4.33; 4.35-4.95; 4.97-5.62; ≥5.65 | 8.0 years | Sex, age, SBP, proteinuria | 8 |
| ATBC 1999(13) | Finland | 28,519 | 50-69 | 100.0 | TC, HDL | HS (197); IS (807) | RR | TC: ≤4.9; 5.0-5.9; 6.0-6.9; ≥7.0/HDL: ≤0.84; 0.85-1.14; 1.15-1.44;  ≥1.45 | 8.0 years | Age, BMI, SBP, serum TC, smoking, alcohol intake, DM, heart disease, education, PA, and tocopherol and carotene supplementation | 8 |
| Renfrew/Paisley 2000(14) | UK | 15,267 | 45-64 | 46.0 | TC | HS (90); IS (236) | RR | ≤5.33; 5.34-5.84; 5.85-6.35;  6.36-6.98; ≥6.99 | 20.0 years | Age, sex, DBP, height, smoking, and  pre-existing CHD | 9 |
| KMIC 2001(15) | Korea | 114,793 | 35-59 | 100.0 | TC | HS (528) | RR | <4.31; 4.31-4.74; 4.74-5.16; 5.16-5.69; ≥5.69 | 6.0 years | Age, smoking, alcohol intake, blood pressure, BMI and blood glucose while fasting | 8 |
| BIP 2001(16) | US | 11,177 | 40-74 | 78.0 | TG | IS (487) | OR | Higher versus lowest | 6.0-8.0 years | Age, sex, lipid-lowering medications, previous MI, DM, hypertension, smoking, PVD, and BMI | 8 |
| Framingham 2002(17) | US | 1,216 | 45-68 | 100.0 | TC | IS (89); HS (18) | RR | Per 40 mg/dL increase | 20.0 years | Age, DBP, serum UA, cigarettes/d, and alcohol intake | 9 |
| Engström 2002(18) | Sweden | 6,063 | 28-61 | 100.0 | TC | IS (200); HS (38) | RR | <4.5; 4.5-5.49; 5.5-6.49; ≥6.5 | 18.7 years | Age | 8 |
| The Oyabe Study 2003(19) | Japan | 4,989 | 35-79 | 30.5 | TC, HDL | IS (35) | RR | TC: <4.16; 4.16-4.68; 4.68-5.20; 5.20-5.72; >5.72/HDL: <0.78; 0.78-1.04; 1.04-1.30; 1.30-1.56; >1.56 | 10.0 years | Sex, age, BMI, SBP, serum TC, cigarette smoking, and alcohol intake | 7 |
| Zhang 2004(20) | China | 5,092 | 18-74 | 100.0 | TC | IS (70); HS (48) | RR | <4.5; 4.5-5.0; 5.0-5.5; 5.5-6.0; >6.0 | 13.5 years | Age, BP, BMI, and smoking | 8 |
| Jood 2004(21) | Sweden | 7,402 | 47-55 | 100.0 | TC | IS (495); HS (144) | HR | <5; 5-6.3; 6.4-7.4; >7.4 | 28.0 years | Age | 8 |
| CHS 2004(22) | US | 4,885 | > 65.0 | 40.0 | TC, TG, LDL, HDL | IS (196); HS (55) | HR | Higher versus lowest | 7.5 years | Age, sex, DM, smoking, CVD, and SBP | 8 |
| Ebrahim 2006(23) | Korea | 787,442 | 30-64 | 84.0 | TC | IS (5,676); HS (3,345) | HR | <3.36; 3.36-4.14; 4.14-5.17; 5.17-6.21; 6.21-6.98; ≥6.98 | 11.0 years | Age, sex, BMI, height, serum glucose, hypertension, ethanol consumption, smoking, PA, monthly pay, and area of residence | 9 |
| AMORIS 2006(24) | Sweden | 175,553 | 20-80 | 56.2 | TC, LDL, HDL, non-HDL | IS (380) | RR | Per SD increase | 10.3 years | Age, gender | 7 |
| Wiberg 2006(25) | Sweden | 2,322 | > 25.0 | 82.0 | TG, LDL, HDL | IS (308); HS (86) | HR | Per SD increase | 32.0 years | Antihypertensive, antidiabetic and lipid-lowering drugs, hypertension, DM, AF, CVD, the metabolicsyndrome, serum cholesterol, smoking, and PA | 8 |
| Sturgeon 2007(26) | US | 21,680 | > 45 | 44.0 | TC, TG, HDL, LDL | HS (135) | RR | 1st Quartile; 2st Quartile; 3st Quartile; 4st Quartile | 15.0 years | Age | 8 |
| WHS 2007(27, 28) | US | 27,937 | > 45 | 0.0 | TC, LDL, HDL, non-HDL | IS (282); HS (137) | HR for IS and RR for HS | TC: < 4.63; 4.63-5.13; 5.13-5.64; 5.64-6.28; >6.28/LDL: <2.48; 2.48-2.92; 2.92-3.34; 3.34-3.88; >3.88/HDL: <1.06;1.06-1.24; 1.24-1.42; 1.42-1.66; >1.66/non-HDL: <3.18; 3.18-3.72; 3.72-4.24; 4.24-4.89; >4.89 | 11.0 for IS, and 19.3 for HS | Age, BMI, alcohol intake, exercise, smoking, history of DM, family history of MI <60 years, treatment for elevated TC, postmenopausal hormone use, migraine status, and randomized treatment assignments, SBP, and antihypertensive treatment | 9 |
| Noda 2009(29) | Japan | 91,219 | 40-79 | 34.0 | TC, TG, LDL, HDL, non-HDL | HS (264) | HR | TC: <4.13; 4.13-4.64; 4.65-5.16; 5.17-5.67; 5.68-6.19/LDL: 2.06; 2.06-2.57; 2.58-3.09;  3.10-3.61; ≥3.62/HDL: 1.03; 1.03-1.28; 1.29- 1.54; 1.55-1.80; ≥1.81/ non-HDL: <2.56; 2.59- 3.08; 3.10-3.59;3.62-4.11; 4.14-4.63; >4.63/TG: < 1.12; 1.12-1.68; 1.69- 2.24; 2.25-2.81; 2.82-3.37; >3.37 | 10.0 years | Age, sex, BP categories, antihypertensive medication use, DM, lipid medicationuse, BMI, glutamyl transferase, smoking, alcohol intake, and kindney dysfunction | 9 |
| JPHC 2009(30, 31) | Japan | 33,469 | 40-69 | 35.0 | TC, TG, HDL | IS (354), HS (207) | HR | TC: <4.65; 4.65-5.16; 5.17-5.68; 5.69-6.20; >6.20/TG: Lowest vs higher/HDL: Lowest vs higher | 12.0 years | Age, study area, time since last meal, TC levels, smoking and ethanol intake | 9 |
| The Hisayama Study 2009(32, 33) | Japan | 2,351 | > 40 | 43.1 | LDL, non-HDL | IS (191), HS (80) | HR | LDL: <2.65; 2.65-3.24; 3.24-3.88; >3.88/non-HDL: <3.14; 3.14-3.77; 3.77-4.49; >4.49 | 19.0 years | Age, sex, HDL cholesterol, TG, SBP, ECG abnormalities, FBG, BMI, current drinking, smoking, and regular exercise | 9 |
| The Suita Study 2009(34, 35) | Japan | 5,098 | 30-79 | 47.2 | TG, HDL, non-HDL | IS (116) | HR | TG: Lowest vs higher/HDL: Lowest vs higher | 11.7 years | Age, BMI, hypertension, DM, HDL cholesterol, smoking and alcohol intake | 7 |
| NOMAS 2009(36) | US | 2,940 | > 39.0 | 36.5 | TC, TG, LDL, HDL, non-HDL | IS (160) | HR | Lowest vs higher | 7.5 years | Age, sex, race/ethnicity, education, hypertension, DM, other lipid profile parameters, smoking, CAD, moderate alcohol intake, and PA | 8 |
| JALS-ECC 2010(37) | Japan | 22,430 | 40-89 | 39.9 | TC, non-HDL | IS (224); HS (113) | RR | TC: <4.558; 4.558-5.128; 5.128-5.776; >5.776/non-HDL: <3.03; 3.03-3.652; 3.652-4.299; >4.299 | 7.6 years | Sex, age, BMI, serum HDL, BP, DM, and smoking | 8 |
| The Rotterdam Study 2011(38) | The Netherlands | 5,773 | >55 | 67.0 | HDL. LDL, TG | HS (85) | HR | HDL: 0.4-1.1; 1.1-1.3; 1.3-1.6; 1.6-5.5/LDL: 0.1-3.2; 3.2-3.7; 3.7-4.3; 4.3-7.9/TG: 0.4-1.0; 1.0-1.3; 1.3-1.8; 1.8-4.3 | 15.0 years | Age, sex, lipid-lowering medication use, SBP, BP-lowering medication use, DM, serum glucose level, serum insulin level, LDL, TG, smoking, BMI, antithrombotic use, and alcohol intake | 9 |
| Copenhagen City Heart Study 2011(39) | Denmark | 13,951 | 54.0 | 45.7 | TC, TG | IS (1,674) | HR | TC: <5; 5-5.99; 6-6.99; 7-7.99; 8-8.99; >9/TG: <1; 1-1.99; 2-2.99; 3-3.99; 4-4.99; >5 | 26.0 years | Age, gender, hypertension, smoking, alcohol intake, AF, lipid lowering therapy, and in women also HRT and menopausal status | 7 |
| Iwate-KENCO 2011(40) | Japan | 24,566 | >18.0 | 35.5 | TC, LDL, HDL | IS (182) | HR | TC: <180; 180-200; 200-220; >220/LDL: <100; 100-120; 120-140; >140/HDL: <50; 50-60; 60-70; >70 | 2.7 years | Age, smoking, SBP, BMI, UA, HbAic | 8 |
| The Jichi Medical School Cohort Study 2011(41, 42) | Japan | 12,334 | 40-69 | 39.2 | TC, non-HDL | Fatal IS (36), HS (26) | HR | TC: <4.14; 4.14-5.16; 5.17-6.21; >6.21/non-HDL: <3.08; 3.08-3.85; >3.85 | 13.0 years | Age, SBP, HDL, smoking, drinking, and BMI | 9 |
| Zhang 2012(43) | Finland | 58,235 | 25-74 | 48.0 | TC, HDL | IS (3,085), HS (829) | HR | TC: <5; 5-5.9; 6-6.9; ≥7.0/HDL: <1; 1.0-1.19; 1.2-1.39; ≥1.4 | 20.1 years | Age, study year, education, PA, smoking, alcohol intake, family history of stroke, BMI, SBP, history of DM, using of cholesterol- lowering agent | 9 |
| HUNT and the Tromsø Study 2012(44) | Norway | 92,408 | >20 | 47.0 | TC, TG, HDL | HS (122) | HR | TC: Per 1.25 mmol/L increase; TG: Lowest vs higher/HDL: Lowest vs higher | 11.0 years | Sex, age, smoking, and alcohol intake | 7 |
| SMART 2013(45) | The Netherlands | 5,731 | 61.0 | 74.1 | TG | IS (193) | HR | <0.97; 0.97-1.24; 1.25-1.60; 1.61-2.24; >2.24 | 4.9 years | Age, gender, BMI, smoking, lipid-lowering medication and LDL | 7 |
| National FINRISK 2013(46, 47) | Finland | 64,349 | 25-74 | 48.3 | TC | HS (437) | HR | <4.92; 4.92-5.58; 5.58-6.22; 6.22-7.06; >7.06 | 17.9 years | Age, sex, SBP, previous MI, smoking, alcohol, and premature stroke in mother | 7 |
| The Kailuan study* 2013(48, 49) | China | 95,778 | 18-98 | 79.5 | LDL, non-HDL, TC, TG, HDL | IS (1,153), HS (753) | HR | LDL: <1.3; 1.3-1.7; 1.7-2.5; 2.5-3.3; 3.3-4.0; >4.0/non-HDL: <2.24; 2.24-2.91; 2.91-3.37; 3.37-3.86; 3.86-4.65 | 4.0 years for IS and 9.0 years for HS | Age, sex, BMI, hypertension, DM, HDL, TG, smoking, drinking, and PA | 9 |
| TLGS 2013(50) | Iran | 2,620 | >50.0 | 46.0 | LDL, non-HDL, TC, TG, HDL | IS (73) | HR | Per 1 mmol/L increase | 9.1 years | Age, (sex for All), SBP, waist to hip ratio, anti-hypertension drug, DM, smoking and lipid lowering drug | 8 |
| CIRCS 2014(51) | Japan | 10,659 | 40-69 | 40.0 | TG | IS (666) | HR | Lowest vs higher | 22.0 years | Age, sex, community, BMI, SBP, use of antihypertensive medication, serum TC, smoking, alcohol intake, serum glucose category, time since last meal and for women, menopausal status | 7 |
| MESA 2015(52) | US | 6,769 | 45-84 | 47.0 | TC, TG, HDL | IS (147) | HR | Per 1 mmol/L increase | 9.5 years | Age, sex, race/ethnicity, education, cigarette, medications, BMI, SBP | 8 |
| REGARDS 2016(53) | US | 24,898 | 64.7 | 44.9 | LDL, HDL, TC, TG, non-HDL | IS (874), HS (77) | HR | Per SD increase | 7.5 years | Age, race, age*race, gender, education, region, income, SBP, hypertensive medication use, lipid lowering medication use, smoking, AF, left ventricular hypertrophy, CVD, and DM | 7 |
| EPIC-Norfolk 2016(54) | UK | 21,798 | 45-79 | 43.9 | LDL | IS (268), HS (117) | HR | <3.24; 3.25-3.88; 3.89-4.59; >4.60 | 12.1 years | Age, sex, smoking, BMI, DM, HDL-c, and SBP | 9 |
| NHIS 2017(55) | Korea | 503,340 | 40-80 | 54.3 | TC | IS (2,082), HS (2,362) | HR | Per 1 mmol/L increase | 10.4 years | Age at baseline, smoking, alcohol intake, PA, BMI, SBP, and FBG | 7 |
| The Strong Heart Study 2017(56) | US | 3,216 | 45-74 | 40.0 | TG, HDL | IS (158) | HR | TG: > 1.70; HDL: <1.03 for men and 1.29 for women | 17.7 years | Age, BMI, smoking, estimated LDL, DM, antihypertensive medications, PA, UACR, and eGFR-MDRD | 8 |
| China Multi-Provincial Cohort 2018(57) | China | 20,954 | 35-64 | 51.5 | LDL | HS (253) | HR | <1.81 | 20.0 years | Age, sex, BMI, smoking, alcohol intake, family history of CVD, FBG, low HDL status, and lipid-lowering medication | 7 |
| Copenhagen General Population Study 2018(58) | Denmark | 106,412 | 58.0 | 45.0 | LDL | IS (2,823) | HR | <2.99; 3-3.99; 4-4.99; >5 | 38.0 years | Age, sex, lipid-lowering therapy, and date of birth | 7 |
| Zheng 2019(59) | China | 5,097 | >35.0 | 43.8 | TC, TG, HDL, LDL, non-HDL | IS (310), HS (187) | HR | TC: <4.52; 4.52-5.17; 5.17-5.84; >5.84/TG: <0.93; 0.93-1.32; 1.32-1.98; >1.98/HDL: <1.20; 1.20-1.40; 1.40-1.98; >1.98/LDL: <2.29; 2.29-2.73; 2.73-3.22; >3.22/non-HDL: <3.23; 3.23-3.76; 3.76-4.29; >4.29 | 8.4 years | Age, sex, ethnicity, BMI, smoking, heavy drinking, DM, SBP, DBP, and anti- hypertension drug treatment | 7 |
| Gu 2019(60) | China | 267,500 | >20.0 | 59.6 | TC, TG, HDL, LDL | IS (5,458), HS (2,186) | HR | TC: <3.108; 3.108-4.144; 4.144-5.18; 5.18-6.216; >6.216/TG: <0.565; 1.13-1.695; 1.695-2.26; >2.26/LDL: 1.813; 1.813-2.59; 2.59-3.367; 3.367-4.144; >4.144/HDL: <1.036; 1.036-1.295; 1.295-1.554; 1.554-1.813; >1.813 | 6.0-19.0 years | Sex, age, smoking, hypertension, geographic region, alcohol intake, education level, and BMI | 7-9 |

*The study abstracted the associations of non-HDL with the risk of IS

1. Okada H, Horibe H, Yoshiyuki O, Hayakawa N, Aoki N. A prospective study of cerebrovascular disease in Japanese rural communities, Akabane and Asahi. Part 1: evaluation of risk factors in the occurrence of cerebral hemorrhage and thrombosis. *Stroke* (1976) 7(6):599-607. doi: 10.1161/01.str.7.6.599.

2. Yano K, Reed DM, MacLean CJ. Serum cholesterol and hemorrhagic stroke in the Honolulu Heart Program. *Stroke* (1989) 20(11):1460-5. doi: 10.1161/01.str.20.11.1460.

3. Iso H, Jacobs DR, Jr., Wentworth D, Neaton JD, Cohen JD. Serum cholesterol levels and six-year mortality from stroke in 350,977 men screened for the multiple risk factor intervention trial. *The New England journal of medicine* (1989) 320(14):904-10. doi: 10.1056/NEJM198904063201405.

4. Neaton JD, Wentworth DN, Cutler J, Stamler J, Kuller L. Risk factors for death from different types of stroke. Multiple Risk Factor Intervention Trial Research Group. *Annals of epidemiology* (1993) 3(5):493-9. doi: 10.1016/1047-2797(93)90103-b.

5. Knekt P, Reunanen A, Aho K, Heliovaara M, Rissanen A, Aromaa A, et al. Risk factors for subarachnoid hemorrhage in a longitudinal population study. *Journal of clinical epidemiology* (1991) 44(9):933-9. doi: 10.1016/0895-4356(91)90056-f.

6. Gatchev O, Rastam L, Lindberg G, Gullberg B, Eklund GA, Isacsson SO. Subarachnoid hemorrhage, cerebral hemorrhage, and serum cholesterol concentration in men and women. *Annals of epidemiology* (1993) 3(4):403-9. doi: 10.1016/1047-2797(93)90068-f.

7. Iribarren C, Jacobs DR, Sadler M, Claxton AJ, Sidney S. Low total serum cholesterol and intracerebral hemorrhagic stroke: is the association confined to elderly men? The Kaiser Permanente Medical Care Program. *Stroke* (1996) 27(11):1993-8. doi: 10.1161/01.str.27.11.1993.

8. Tanne D, Yaari S, Goldbourt U. High-density lipoprotein cholesterol and risk of ischemic stroke mortality. A 21-year follow-up of 8586 men from the Israeli Ischemic Heart Disease Study. *Stroke* (1997) 28(1):83-7. doi: 10.1161/01.str.28.1.83.

9. Howard G, Wagenknecht LE, Cai J, Cooper L, Kraut MA, Toole JF. Cigarette smoking and other risk factors for silent cerebral infarction in the general population. *Stroke* (1998) 29(5):913-7. doi: 10.1161/01.str.29.5.913.

10. Shahar E, Chambless LE, Rosamond WD, Boland LL, Ballantyne CM, McGovern PG, et al. Plasma lipid profile and incident ischemic stroke: the Atherosclerosis Risk in Communities (ARIC) study. *Stroke* (2003) 34(3):623-31. doi: 10.1161/01.STR.0000057812.51734.FF.

11. Ohira T, Shahar E, Chambless LE, Rosamond WD, Mosley TH, Jr., Folsom AR. Risk factors for ischemic stroke subtypes: the Atherosclerosis Risk in Communities study. *Stroke* (2006) 37(10):2493-8. doi: 10.1161/01.STR.0000239694.19359.88.

12. Okumura K, Iseki K, Wakugami K, Kimura Y, Muratani H, Ikemiya Y, et al. Low serum cholesterol as a risk factor for hemorrhagic stroke in men: a community-based mass screening in Okinawa, Japan. *Japanese circulation journal* (1999) 63(1):53-8. doi: 10.1253/jcj.63.53.

13. Leppala JM, Virtamo J, Fogelholm R, Albanes D, Heinonen OP. Different risk factors for different stroke subtypes: association of blood pressure, cholesterol, and antioxidants. *Stroke* (1999) 30(12):2535-40. doi: 10.1161/01.str.30.12.2535.

14. Hart CL, Hole DJ, Smith GD. The relation between cholesterol and haemorrhagic or ischaemic stroke in the Renfrew/Paisley study. *J Epidemiol Community Health* (2000) 54(11):874-5. doi: 10.1136/jech.54.11.874.

15. Suh I, Jee SH, Kim HC, Nam CM, Kim IS, Appel LJ. Low serum cholesterol and haemorrhagic stroke in men: Korea Medical Insurance Corporation Study. *Lancet* (2001) 357(9260):922-5. doi: 10.1016/S0140-6736(00)04213-6.

16. Tanne D, Koren-Morag N, Graff E, Goldbourt U. Blood lipids and first-ever ischemic stroke/transient ischemic attack in the Bezafibrate Infarction Prevention (BIP) Registry: high triglycerides constitute an independent risk factor. *Circulation* (2001) 104(24):2892-7. doi: 10.1161/hc4901.100384.

17. Rodriguez BL, D'Agostino R, Abbott RD, Kagan A, Burchfiel CM, Yano K, et al. Risk of hospitalized stroke in men enrolled in the Honolulu Heart Program and the Framingham Study: A comparison of incidence and risk factor effects. *Stroke* (2002) 33(1):230-6. doi: 10.1161/hs0102.101081.

18. Engstrom G, Lind P, Hedblad B, Stavenow L, Janzon L, Lindgarde F. Effects of cholesterol and inflammation-sensitive plasma proteins on incidence of myocardial infarction and stroke in men. *Circulation* (2002) 105(22):2632-7. doi: 10.1161/01.cir.0000017327.69909.ff.

19. Soyama Y, Miura K, Morikawa Y, Nishijo M, Nakanishi Y, Naruse Y, et al. High-density lipoprotein cholesterol and risk of stroke in Japanese men and women: the Oyabe Study. *Stroke* (2003) 34(4):863-8. doi: 10.1161/01.STR.0000060869.34009.38.

20. Zhang XF, Attia J, D'Este C, Yu XH. Prevalence and magnitude of classical risk factors for stroke in a cohort of 5092 Chinese steelworkers over 13.5 years of follow-up. *Stroke* (2004) 35(5):1052-6. doi: 10.1161/01.STR.0000125305.12859.ff.

21. Jood K, Jern C, Wilhelmsen L, Rosengren A. Body mass index in mid-life is associated with a first stroke in men: a prospective population study over 28 years. *Stroke* (2004) 35(12):2764-9. doi: 10.1161/01.STR.0000147715.58886.ad.

22. Psaty BM, Anderson M, Kronmal RA, Tracy RP, Orchard T, Fried LP, et al. The association between lipid levels and the risks of incident myocardial infarction, stroke, and total mortality: The Cardiovascular Health Study. *Journal of the American Geriatrics Society* (2004) 52(10):1639-47. doi: 10.1111/j.1532-5415.2004.52455.x.

23. Ebrahim S, Sung J, Song YM, Ferrer RL, Lawlor DA, Davey Smith G. Serum cholesterol, haemorrhagic stroke, ischaemic stroke, and myocardial infarction: Korean national health system prospective cohort study. *Bmj* (2006) 333(7557):22. doi: 10.1136/bmj.38855.610324.80.

24. Walldius G, Aastveit AH, Jungner I. Stroke mortality and the apoB/apoA-I ratio: results of the AMORIS prospective study. *Journal of internal medicine* (2006) 259(3):259-66. doi: 10.1111/j.1365-2796.2005.01610.x.

25. Wiberg B, Sundstrom J, Arnlov J, Terent A, Vessby B, Zethelius B, et al. Metabolic risk factors for stroke and transient ischemic attacks in middle-aged men: a community-based study with long-term follow-up. *Stroke* (2006) 37(12):2898-903. doi: 10.1161/01.STR.0000249056.24657.8b.

26. Sturgeon JD, Folsom AR, Longstreth WT, Jr., Shahar E, Rosamond WD, Cushman M. Risk factors for intracerebral hemorrhage in a pooled prospective study. *Stroke* (2007) 38(10):2718-25. doi: 10.1161/STROKEAHA.107.487090.

27. Kurth T, Everett BM, Buring JE, Kase CS, Ridker PM, Gaziano JM. Lipid levels and the risk of ischemic stroke in women. *Neurology* (2007) 68(8):556-62. doi: 10.1212/01.wnl.0000254472.41810.0d.

28. Rist PM, Buring JE, Ridker PM, Kase CS, Kurth T, Rexrode KM. Lipid levels and the risk of hemorrhagic stroke among women. *Neurology* (2019) 92(19):e2286-e94. doi: 10.1212/WNL.0000000000007454.

29. Noda H, Iso H, Irie F, Sairenchi T, Ohtaka E, Doi M, et al. Low-density lipoprotein cholesterol concentrations and death due to intraparenchymal hemorrhage: the Ibaraki Prefectural Health Study. *Circulation* (2009) 119(16):2136-45. doi: 10.1161/CIRCULATIONAHA.108.795666.

30. Noda H, Iso H, Saito I, Konishi M, Inoue M, Tsugane S, et al. The impact of the metabolic syndrome and its components on the incidence of ischemic heart disease and stroke: the Japan public health center-based study. *Hypertension research : official journal of the Japanese Society of Hypertension* (2009) 32(4):289-98. doi: 10.1038/hr.2009.14.

31. Cui R, Iso H, Yamagishi K, Saito I, Kokubo Y, Inoue M, et al. High serum total cholesterol levels is a risk factor of ischemic stroke for general Japanese population: the JPHC study. *Atherosclerosis* (2012) 221(2):565-9. doi: 10.1016/j.atherosclerosis.2012.01.013.

32. Imamura T, Doi Y, Arima H, Yonemoto K, Hata J, Kubo M, et al. LDL cholesterol and the development of stroke subtypes and coronary heart disease in a general Japanese population: the Hisayama study. *Stroke* (2009) 40(2):382-8. doi: 10.1161/STROKEAHA.108.529537.

33. Imamura T, Doi Y, Ninomiya T, Hata J, Nagata M, Ikeda F, et al. Non-high-density lipoprotein cholesterol and the development of coronary heart disease and stroke subtypes in a general Japanese population: the Hisayama Study. *Atherosclerosis* (2014) 233(2):343-8. doi: 10.1016/j.atherosclerosis.2014.01.005.

34. Okamura T, Kokubo Y, Watanabe M, Higashiyama A, Ono Y, Miyamoto Y, et al. Triglycerides and non-high-density lipoprotein cholesterol and the incidence of cardiovascular disease in an urban Japanese cohort: the Suita study. *Atherosclerosis* (2010) 209(1):290-4. doi: 10.1016/j.atherosclerosis.2009.09.012.

35. Okamura T, Kokubo Y, Watanabe M, Higashiyama A, Ono Y, Nishimura K, et al. A revised definition of the metabolic syndrome predicts coronary artery disease and ischemic stroke after adjusting for low density lipoprotein cholesterol in a 13-year cohort study of Japanese: the Suita study. *Atherosclerosis* (2011) 217(1):201-6. doi: 10.1016/j.atherosclerosis.2011.03.010.

36. Willey JZ, Xu Q, Boden-Albala B, Paik MC, Moon YP, Sacco RL, et al. Lipid profile components and risk of ischemic stroke: the Northern Manhattan Study (NOMAS). *Archives of neurology* (2009) 66(11):1400-6. doi: 10.1001/archneurol.2009.210.

37. Tanabe N, Iso H, Okada K, Nakamura Y, Harada A, Ohashi Y, et al. Serum total and non-high-density lipoprotein cholesterol and the risk prediction of cardiovascular events - the JALS-ECC. *Circulation journal : official journal of the Japanese Circulation Society* (2010) 74(7):1346-56. doi: 10.1253/circj.cj-09-0861.

38. Wieberdink RG, Poels MM, Vernooij MW, Koudstaal PJ, Hofman A, van der Lugt A, et al. Serum lipid levels and the risk of intracerebral hemorrhage: the Rotterdam Study. *Arteriosclerosis, thrombosis, and vascular biology* (2011) 31(12):2982-9. doi: 10.1161/ATVBAHA.111.234948.

39. Varbo A, Nordestgaard BG, Tybjaerg-Hansen A, Schnohr P, Jensen GB, Benn M. Nonfasting triglycerides, cholesterol, and ischemic stroke in the general population. *Annals of neurology* (2011) 69(4):628-34. doi: 10.1002/ana.22384.

40. Yokokawa H, Yasumura S, Tanno K, Ohsawa M, Onoda T, Itai K, et al. Serum low-density lipoprotein to high-density lipoprotein ratio as a predictor of future acute myocardial infarction among men in a 2.7-year cohort study of a Japanese northern rural population. *Journal of atherosclerosis and thrombosis* (2011) 18(2):89-98. doi: 10.5551/jat.5215.

41. Nago N, Ishikawa S, Goto T, Kayaba K. Low cholesterol is associated with mortality from stroke, heart disease, and cancer: the Jichi Medical School Cohort Study. *Journal of epidemiology* (2011) 21(1):67-74. doi: 10.2188/jea.je20100065.

42. Kakehi E, Kotani K, Ishikawa S, Gotoh T, Kayaba K, Nakamura Y, et al. Serum non-high-density lipoprotein cholesterol levels and the incidence of ischemic stroke in a Japanese population: the Jichi Medical School cohort study. *Asia-Pacific journal of public health* (2015) 27(2):NP535-43. doi: 10.1177/1010539513475649.

43. Zhang Y, Tuomilehto J, Jousilahti P, Wang Y, Antikainen R, Hu G. Total and high-density lipoprotein cholesterol and stroke risk. *Stroke* (2012) 43(7):1768-74. doi: 10.1161/STROKEAHA.111.646778.

44. Sandvei MS, Lindekleiv H, Romundstad PR, Muller TB, Vatten LJ, Ingebrigtsen T, et al. Risk factors for aneurysmal subarachnoid hemorrhage - BMI and serum lipids: 11-year follow-up of the HUNT and the Tromso Study in Norway. *Acta neurologica Scandinavica* (2012) 125(6):382-8. doi: 10.1111/j.1600-0404.2011.01578.x.

45. van de Woestijne AP, Wassink AM, Monajemi H, Liem AH, Nathoe HM, van der Graaf Y, et al. Plasma triglyceride levels increase the risk for recurrent vascular events independent of LDL-cholesterol or nonHDL-cholesterol. *International journal of cardiology* (2013) 167(2):403-8. doi: 10.1016/j.ijcard.2012.01.008.

46. Korja M, Silventoinen K, Laatikainen T, Jousilahti P, Salomaa V, Hernesniemi J, et al. Risk factors and their combined effects on the incidence rate of subarachnoid hemorrhage--a population-based cohort study. *PloS one* (2013) 8(9):e73760. doi: 10.1371/journal.pone.0073760.

47. Lindbohm J, Korja M, Jousilahti P, Salomaa V, Kaprio J. Adverse lipid profile elevates risk for subarachnoid hemorrhage: A prospective population-based cohort study. *Atherosclerosis* (2018) 274:112-9. doi: 10.1016/j.atherosclerosis.2018.05.011.

48. Ma C, Gurol ME, Huang Z, Lichtenstein AH, Wang X, Wang Y, et al. Low-density lipoprotein cholesterol and risk of intracerebral hemorrhage: A prospective study. *Neurology* (2019) 93(5):e445-e57. doi: 10.1212/WNL.0000000000007853.

49. Wu J, Chen S, Liu L, Gao X, Zhou Y, Wang C, et al. Non-high-density lipoprotein cholesterol vs low-density lipoprotein cholesterol as a risk factor for ischemic stroke: a result from the Kailuan study. *Neurological research* (2013) 35(5):505-11. doi: 10.1179/1743132813Y.0000000206.

50. Tohidi M, Mohebi R, Cheraghi L, Hajsheikholeslami F, Aref S, Nouri S, et al. Lipid profile components and incident cerebrovascular events versus coronary heart disease; the result of 9 years follow-up in Tehran Lipid and Glucose Study. *Clinical biochemistry* (2013) 46(9):716-21. doi: 10.1016/j.clinbiochem.2013.03.012.

51. Iso H, Imano H, Yamagishi K, Ohira T, Cui R, Noda H, et al. Fasting and non-fasting triglycerides and risk of ischemic cardiovascular disease in Japanese men and women: the Circulatory Risk in Communities Study (CIRCS). *Atherosclerosis* (2014) 237(1):361-8. doi: 10.1016/j.atherosclerosis.2014.08.028.

52. Reina SA, Llabre MM, Allison MA, Wilkins JT, Mendez AJ, Arnan MK, et al. HDL cholesterol and stroke risk: The Multi-Ethnic Study of Atherosclerosis. *Atherosclerosis* (2015) 243(1):314-9. doi: 10.1016/j.atherosclerosis.2015.09.031.

53. Glasser SP, Mosher A, Howard G, Banach M. What is the association of lipid levels and incident stroke? *International journal of cardiology* (2016) 220:890-4. doi: 10.1016/j.ijcard.2016.06.091.

54. Stoekenbroek RM, Boekholdt SM, Luben R, Hovingh GK, Zwinderman AH, Wareham NJ, et al. Heterogeneous impact of classic atherosclerotic risk factors on different arterial territories: the EPIC-Norfolk prospective population study. *European heart journal* (2016) 37(11):880-9. doi: 10.1093/eurheartj/ehv630.

55. Yi SW, Shin DH, Kim H, Yi JJ, Ohrr H. Total cholesterol and stroke mortality in middle-aged and elderly adults: A prospective cohort study. *Atherosclerosis* (2018) 270:211-7. doi: 10.1016/j.atherosclerosis.2017.12.003.

56. Lee JS, Chang PY, Zhang Y, Kizer JR, Best LG, Howard BV. Triglyceride and HDL-C Dyslipidemia and Risks of Coronary Heart Disease and Ischemic Stroke by Glycemic Dysregulation Status: The Strong Heart Study. *Diabetes care* (2017) 40(4):529-37. doi: 10.2337/dc16-1958.

57. Zhang X, Liu J, Wang M, Qi Y, Sun J, Liu J, et al. Twenty-year epidemiologic study on LDL-C levels in relation to the risks of atherosclerotic event, hemorrhagic stroke, and cancer death among young and middle-aged population in China. *Journal of clinical lipidology* (2018) 12(5):1179-89 e4. doi: 10.1016/j.jacl.2018.06.011.

58. Beheshti S, Madsen CM, Varbo A, Benn M, Nordestgaard BG. Relationship of Familial Hypercholesterolemia and High Low-Density Lipoprotein Cholesterol to Ischemic Stroke: Copenhagen General Population Study. *Circulation* (2018) 138(6):578-89. doi: 10.1161/CIRCULATIONAHA.118.033470.

59. Zheng J, Sun Z, Zhang X, Li Z, Guo X, Xie Y, et al. Non-traditional lipid profiles associated with ischemic stroke not hemorrhagic stroke in hypertensive patients: results from an 8.4 years follow-up study. *Lipids in health and disease* (2019) 18(1):9. doi: 10.1186/s12944-019-0958-y.

60. Gu X, Li Y, Chen S, Yang X, Liu F, Li Y, et al. Association of Lipids With Ischemic and Hemorrhagic Stroke: A Prospective Cohort Study Among 267 500 Chinese. *Stroke* (2019) 50(12):3376-84. doi: 10.1161/STROKEAHA.119.026402.
