# Supplementary material for: Associations of lipid profiles with the risk of ischemic and hemorrhagic stroke: A systematic review and meta-analysis of prospective cohort studies
Source: Front Cardiovasc Med. 2022 Nov 3;9:893248. doi: 10.3389/fcvm.2022.893248 (PMC9668898; doi:10.3389/fcvm.2022.893248)
Supplement: Supplementary file 1 [file Data_Sheet_1.ZIP › 893248_SupMaterial/supplementary Table 2.DOCX]

**supplementary Table 2. Subgroup analyses for per 1 mmol/L increase in lipid profiles and the risk of ischemic and hemorrhagic stroke**

| Lipid profiles | Factors | Outcomes | Subgroup | RR and 95%CI | P value | Heterogeneity (%) | P value for heterogeneity | Difference between IS and HS | Difference between subgroups for IS | Difference between subgroups for HS |
| --- | --- | --- | --- | --- | --- | --- | --- | --- | --- | --- |
| TC | Country | Ischemic stroke | Western | 1.06 (1.03-1.09) | <0.001 | 83.3 | <0.001 | <0.001 | 0.574 | 0.055 |
|  |  | Hemorrhagic stroke | Western | 0.98 (0.95-1.00) | 0.055 | 57.3 | 0.001 |  |  |  |
|  |  | Ischemic stroke | Eastern | 1.03 (1.00-1.07) | 0.051 | 83.4 | <0.001 | 0.001 |  |  |
|  |  | Hemorrhagic stroke | Eastern | 0.96 (0.94-0.98) | <0.001 | 39.7 | 0.047 |  |  |  |
|  | Sex | Ischemic stroke | Male | 1.03 (1.01-1.05) | 0.004 | 60.6 | 0.001 | <0.001 | <0.001 | 0.001 |
|  |  | Hemorrhagic stroke | Male | 0.96 (0.94-0.99) | 0.003 | 58.9 | 0.001 |  |  |  |
|  |  | Ischemic stroke | Female | 1.04 (1.00-1.09) | 0.068 | 80.3 | <0.001 | 0.143 |  |  |
|  |  | Hemorrhagic stroke | Female | 1.00 (0.97-1.03) | 0.870 | 48.7 | 0.048 |  |  |  |
|  |  | Ischemic stroke | Both | 1.07 (1.01-1.12) | 0.020 | 90.3 | <0.001 | <0.001 |  |  |
|  |  | Hemorrhagic stroke | Both | 0.94 (0.92-0.96) | <0.001 | 0.0 | 0.795 |  |  |  |
|  | Follow-up duration (years) | Ischemic stroke | ≥ 10.0 | 1.06 (1.04-1.09) | <0.001 | 84.7 | <0.001 | <0.001 | <0.001 | 0.285 |
|  |  | Hemorrhagic stroke | ≥ 10.0 | 0.97 (0.95-0.99) | 0.005 | 58.0 | <0.001 |  |  |  |
|  |  | Ischemic stroke | < 10.0 | 1.01 (0.98-1.03) | 0.589 | 53.1 | 0.015 | 0.006 |  |  |
|  |  | Hemorrhagic stroke | < 10.0 | 0.96 (0.93-0.98) | <0.001 | 22.9 | 0.232 |  |  |  |
|  | Adjusted levels | Ischemic stroke | High | 1.04 (1.02-1.07) | <0.001 | 83.7 | <0.001 | <0.001 | 0.191 | 0.847 |
|  |  | Hemorrhagic stroke | High | 0.97 (0.95-0.99) | <0.001 | 55.3 | <0.001 |  |  |  |
|  |  | Ischemic stroke | Low | 1.09 (1.02-1.16) | 0.009 | 0.0 | 0.374 | <0.001 |  |  |
|  |  | Hemorrhagic stroke | Low | 0.97 (0.93-1.00) | 0.053 | 25.1 | 0.254 |  |  |  |
| TG | Country | Ischemic stroke | Western | 1.09 (1.03-1.16) | 0.004 | 85.5 | <0.001 | <0.001 | 0.005 | <0.001 |
|  |  | Hemorrhagic stroke | Western | 0.76 (0.64-0.91) | 0.002 | 58.0 | 0.049 |  |  |  |
|  |  | Ischemic stroke | Eastern | 1.07 (0.92-1.24) | 0.395 | 7.0 | 0.341 | 0.416 |  |  |
|  |  | Hemorrhagic stroke | Eastern | 1.00 (0.93-1.06) | 0.895 | 0.0 | 0.556 |  |  |  |
|  | Sex | Ischemic stroke | Male | 1.08 (1.03-1.12) | 0.001 | 0.0 | 0.529 | - | <0.001 | 0.042 |
|  |  | Hemorrhagic stroke | Male | - | - | - | - |  |  |  |
|  |  | Ischemic stroke | Female | 1.18 (1.11-1.25) | <0.001 | 0.0 | 0.431 | <0.001 |  |  |
|  |  | Hemorrhagic stroke | Female | 0.73 (0.57-0.93) | 0.012 | - | - |  |  |  |
|  |  | Ischemic stroke | Both | 1.07 (1.01-1.12) | 0.012 | 86.6 | <0.001 | 0.005 |  |  |
|  |  | Hemorrhagic stroke | Both | 0.86 (0.75-1.00) | 0.043 | 75.4 | 0.001 |  |  |  |
|  | Follow-up duration (years) | Ischemic stroke | ≥ 10.0 | 1.10 (1.06-1.13) | <0.001 | 54.4 | 0.052 | 0.001 | <0.001 | 0.148 |
|  |  | Hemorrhagic stroke | ≥ 10.0 | 0.86 (0.74-0.99) | 0.034 | 77.6 | <0.001 |  |  |  |
|  |  | Ischemic stroke | < 10.0 | 1.06 (0.97-1.15) | 0.188 | 57.9 | 0.037 | 0.029 |  |  |
|  |  | Hemorrhagic stroke | < 10.0 | 0.76 (0.57-1.01) | 0.060 | - | - |  |  |  |
|  | Adjusted levels | Ischemic stroke | High | 1.08 (1.04-1.13) | <0.001 | 83.1 | <0.01 | 0.003 | - | 0.001 |
|  |  | Hemorrhagic stroke | High | 0.89 (0.79-1.01) | 0.067 | 64.1 | 0.016 |  |  |  |
|  |  | Ischemic stroke | Low | - | - | - | - | - |  |  |
|  |  | Hemorrhagic stroke | Low | 0.67 (0.54-0.83) | <0.001 | - | - |  |  |  |
| LDL | Country | Ischemic stroke | Western | 1.03 (1.00-1.07) | 0.052 | 55.5 | 0.021 | 0.243 | 0.026 | 0.368 |
|  |  | Hemorrhagic stroke | Western | 0.98 (0.91-1.06) | 0.612 | 47.0 | 0.109 |  |  |  |
|  |  | Ischemic stroke | Eastern | 1.02 (0.93-1.11) | 0.725 | 70.3 | 0.005 | 0.316 |  |  |
|  |  | Hemorrhagic stroke | Eastern | 0.94 (0.82-1.07) | 0.337 | 84.8 | 0.001 |  |  |  |
|  | Sex | Ischemic stroke | Male | 0.92 (0.83-1.01) | 0.095 | 0.0 | 0.641 | - | 0.020 | 0.011 |
|  |  | Hemorrhagic stroke | Male | - | - | - | - |  |  |  |
|  |  | Ischemic stroke | Female | 1.11 (0.91-1.36) | 0.313 | 74.4 | 0.020 | 0.743 |  |  |
|  |  | Hemorrhagic stroke | Female | 1.07 (0.98-1.17) | 0.134 | - | - |  |  |  |
|  |  | Ischemic stroke | Both | 1.03 (1.00-1.07) | 0.031 | 65.5 | 0.003 | 0.012 |  |  |
|  |  | Hemorrhagic stroke | Both | 0.94 (0.89-1.01) | 0.073 | 60.2 | 0.020 |  |  |  |
|  | Follow-up duration (years) | Ischemic stroke | ≥ 10.0 | 1.05 (1.02-1.08) | <0.001 | 34.5 | 0.142 | 0.017 | 0.001 | 0.709 |
|  |  | Hemorrhagic stroke | ≥ 10.0 | 0.96 (0.90-1.03) | 0.234 | 71.9 | 0.002 |  |  |  |
|  |  | Ischemic stroke | < 10.0 | 0.99 (0.91-1.08) | 0.824 | 70.6 | 0.004 | 0.855 |  |  |
|  |  | Hemorrhagic stroke | < 10.0 | 1.02 (0.75-1.39) | 0.900 | - | - |  |  |  |
|  | Adjusted levels | Ischemic stroke | High | 1.03 (1.00-1.07) | 0.064 | 67.3 | <0.001 | 0.141 | 0.991 | 0.210 |
|  |  | Hemorrhagic stroke | High | 0.97 (0.90-1.04) | 0.439 | 69.9 | 0.003 |  |  |  |
|  |  | Ischemic stroke | Low | 1.03 (0.93-1.15) | 0.585 | - | - | 0.086 |  |  |
|  |  | Hemorrhagic stroke | Low | 0.91 (0.83-1.00) | 0.047 | - | - |  |  |  |
| HDL | Country | Ischemic stroke | Western | 0.90 (0.82-0.99) | 0.041 | 77.1 | <0.001 | 0.126 | 0.088 | 0.009 |
|  |  | Hemorrhagic stroke | Western | 1.01 (0.90-1.13) | 0.890 | 23.8 | 0.217 |  |  |  |
|  |  | Ischemic stroke | Eastern | 0.86 (0.75-0.99) | 0.041 | 29.8 | 0.212 | 0.865 |  |  |
|  |  | Hemorrhagic stroke | Eastern | 0.88 (0.70-1.10) | 0.264 | 75.5 | 0.043 |  |  |  |
|  | Sex | Ischemic stroke | Male | 0.96 (0.81-1.15) | 0.663 | 79.6 | <0.001 | 0.437 | 0.080 | 0.258 |
|  |  | Hemorrhagic stroke | Male | 0.84 (0.63-1.12) | 0.225 | 52.0 | 0.100 |  |  |  |
|  |  | Ischemic stroke | Female | 0.83 (0.70-0.98) | 0.025 | 37.3 | 0.188 | 0.056 |  |  |
|  |  | Hemorrhagic stroke | Female | 1.09 (0.87-1.36) | 0.468 | 0.0 | 0.443 |  |  |  |
|  |  | Ischemic stroke | Both | 0.88 (0.80-0.96) | 0.007 | 69.0 | 0.004 | 0.222 |  |  |
|  |  | Hemorrhagic stroke | Both | 0.98 (0.85-1.14) | 0.828 | 62.6 | 0.020 |  |  |  |
|  | Follow-up duration (years) | Ischemic stroke | ≥ 10.0 | 0.89 (0.81-0.99) | 0.026 | 74.1 | <0.001 | 0.274 | 0.238 | 0.805 |
|  |  | Hemorrhagic stroke | ≥ 10.0 | 0.97 (0.87-1.10) | 0.672 | 47.5 | 0.046 |  |  |  |
|  |  | Ischemic stroke | < 10.0 | 0.89 (0.78-1.02) | 0.105 | 67.3 | 0.003 | 0.914 |  |  |
|  |  | Hemorrhagic stroke | < 10.0 | 0.91 (0.62-1.33) | 0.632 | 70.3 | 0.034 |  |  |  |
|  | Adjusted levels | Ischemic stroke | High | 0.90 (0.83-0.98) | 0.014 | 69.4 | <0.001 | 0.458 | 0.032 | 0.048 |
|  |  | Hemorrhagic stroke | High | 0.95 (0.84-1.06) | 0.345 | 45.2 | 0.045 |  |  |  |
|  |  | Ischemic stroke | Low | 0.80 (0.72-0.89) | <0.001 | - | - | 0.004 |  |  |
|  |  | Hemorrhagic stroke | Low | 1.17 (0.92-1.48) | 0.195 | - | - |  |  |  |
| Non-HDL | Country | Ischemic stroke | Western | 1.07 (0.97-1.18) | 0.165 | 87.4 | <0.001 | 0.453 | 0.663 | 0.926 |
|  |  | Hemorrhagic stroke | Western | 0.95 (0.71-1.28) | 0.733 | - | - |  |  |  |
|  |  | Ischemic stroke | Eastern | 1.01 (0.94-1.08) | 0.762 | 71.2 | 0.001 | 0.537 |  |  |
|  |  | Hemorrhagic stroke | Eastern | 0.96 (0.83-1.11) | 0.558 | 88.4 | <0.001 |  |  |  |
|  | Sex | Ischemic stroke | Male | 0.95 (0.73-1.23) | 0.697 | 58.9 | 0.119 | - | 0.808 | - |
|  |  | Hemorrhagic stroke | Male | - | - | - | - |  |  |  |
|  |  | Ischemic stroke | Female | 1.09 (0.88-1.36) | 0.439 | 90.2 | <0.001 | - |  |  |
|  |  | Hemorrhagic stroke | Female | - | - | - | - |  |  |  |
|  |  | Ischemic stroke | Both | 1.03 (0.98-1.09) | 0.232 | 76.0 | <0.001 | 0.327 |  |  |
|  |  | Hemorrhagic stroke | Both | 0.96 (0.84-1.09) | 0.500 | 82.7 | 0.001 |  |  |  |
|  | Follow-up duration (years) | Ischemic stroke | ≥ 10.0 | 1.03 (0.95-1.12) | 0.446 | 79.0 | 0.001 | 0.325 | 0981 | 0.009 |
|  |  | Hemorrhagic stroke | ≥ 10.0 | 0.92 (0.75-1.14) | 0.457 | 90.2 | 0.001 |  |  |  |
|  |  | Ischemic stroke | < 10.0 | 1.04 (0.96-1.11) | 0.359 | 79.4 | <0001 | 0.849 |  |  |
|  |  | Hemorrhagic stroke | < 10.0 | 1.03 (0.96-1.10) | 0.452 | 0.0 | 0.600 |  |  |  |
|  | Adjusted levels | Ischemic stroke | High | 1.03 (0.97-1.09) | 0.317 | 78.6 | <0.001 | 0.334 | 0.230 | - |
|  |  | Hemorrhagic stroke | High | 0.96 (0.84-1.09) | 0.500 | 82.7 | 0.001 |  |  |  |
|  |  | Ischemic stroke | Low | 1.10 (0.99-1.22) | 0.074 | - | - | - |  |  |
|  |  | Hemorrhagic stroke | Low | - | - | - | - |  |  |  |

*RRI: relative risk increase; RR: relative risk; CI: confidence interval.
