# Supplementary figures and images for: Associations of lipid profiles with the risk of ischemic and hemorrhagic stroke: A systematic review and meta-analysis of prospective cohort studies
Source: Front Cardiovasc Med. 2022 Nov 3;9:893248. doi: 10.3389/fcvm.2022.893248 (PMC9668898; doi:10.3389/fcvm.2022.893248)

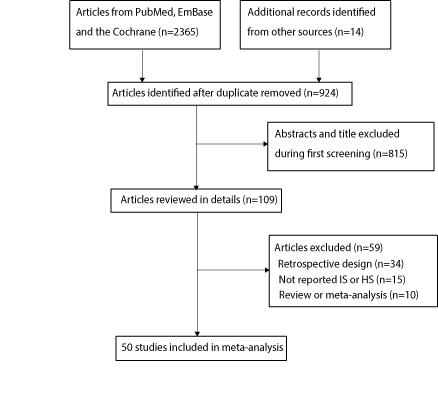


**Figure S1. The flow diagram for literature search and trials selection process.**

Supplement: Supplementary file 1 [file Data_Sheet_1.ZIP › 893248_SupMaterial/S2 File.DOCX]
